# Supplementary material for: Human Herpesvirus 1 Associated with Epizootics in Belo Horizonte, Minas Gerais, Brazil
Source: Viruses. 2025 Apr 30;17(5):660. doi: 10.3390/v17050660 (PMC12115514; doi:10.3390/v17050660)
Supplement: Supplementary file 1 [file viruses-17-00660-s001.zip › viruses-3575738-supplementary.pdf]

Supplementary material - Human herpesvirus 1 associated with epizootics in Belo Horizonte, Minas Gerais, Brazil

Garcia-Oliveira et al.

Table S1: Primers and probes used for molecular investigation of yellow fever virus and human herpesviruses

Target	Primer or probe	Sequence	Region	Reference
YFV	YFallF primer	5'-GCTAATTGAGGTGYATTGGTCTGC-3'	5' UTR	Domingo et al, 2012
	YFallR primer	5'-CTGCTAATCGCTCAAMGAACG-3'		
	YFallP probe	5′-FAM-ATCGAGTTGCTAGGCAATAAACAC-TMR-3′		
HHV-1/HHV-2	HHV-1/2 F	5′-ATCAGGGTAGCCGCGCGTGTGACA-3′	DNA POL	De Oliveira, 2015
	HHV-1/2 R	5′-CATACCGGAACGACCACAC-3′		
HHV-3	HHV-3 F	5′-CATCTGCAATTATGGCTCCAA-3′	DNA POL	De Oliveira, 2015
	HHV-3 R	5′-GTTTCCATTGCTGAAT-3′		
Endogenous control	β-actin F	5'-CCAACCGCGAGAAGATGA-3'	β-actin	Rezende et al, 2019
	β-actin R	5'-CCAGAGGCGTACAGGGATAG-3'		
HHV	Primer KS30	5'-biotinyl-TTCAAGGCCACCATGTACTACAAAGACGT-3'	Glycoprotein B	Orle et al, 1996
	Primer KS31	5'-biotinyl-GCCGTAAAACGGGGACATGTACACAAAGT-3'		
HHV-1	Forward primer (HSV1UP)	5'-CGGCCGTGTGACACTATCG-3'	Glycoprotein D	Weidmann et al, 2003
	Reverse primer (HSV1DP)	5'-CTCGTAAAATGGCCCCTCC-3'		
	Probe (HSV1P)	5'-CCATACCGACCACACCGACGAACC-3'		
HHV-2	Forward primer (HSV2UP)	5'-CGCTCTCGTAAATGCTTCCCT-3'	Glycoprotein G	Weidmann et al, 2003
	Reverse primer (HSV2DP)	5'-TCTACCCACAACAGACCCACG-3'		
	Probe (HSV2P)	5'-CGCGGAGACATTCGAGTACCAGATCG-3'		

YFV: Yellow Fever Virus – *Orthoflavivirus flavi*; HHV: Human Herpesvirus; HHV-1: human alphaherpesvirus 1 - *Simplexvirus humanalpha1*; HHV-2: human alphaherpesvirus 2 - *Simplexvirus humanalpha*2; HHV-3: Varicela-Zoster virus - *Varicellovirus humanalpha3;* 5′UTR: 5' untranslated region; DNA POL: DNA polymerase.

Table S2: Necropsies findings in *Callithrix penicilatta* carcasses

	Marmosets/Macroscopic aspects of necropysis															
	1342				1343				1344				1345			
Sample	Coloration	Size	Consistency	Aspect	Coloration	Size	Consistency	Aspect	Coloration	Size	Consistency	Aspect	Coloration	Size	Consistency	Aspect
brain	na	na	na	na	na	na	na	na	na	na	na	na	na	na	na	na
heart	reddish	enlarged	hardened	rough	normal	normal	soft	smooth	darkened	enlarged	ni	ni	darkened	normal	hardened	normal
lung	normal	normal	soft	smooth	reddish	enlarged	hardened	rough	reddish	enlarged	flabby	hemorrhagic	reddish	normal	flabby	hemorrhagic
liver	yellowish	reduced	flabby	necrotic	darkened	reduced	necrotic	flabby	darkened	enlarged	ni	ni	na	na	na	na
kidney	darkened	enlarged	hardened	granular	normal	normal	soft	smooth	reddish	normal	soft	smooth	anemic	enlarged	soft	normal
spleen	anemic	normal	normal	smooth	greenish	enlarged	flabby	rough	na	na	na	na	na	na	na	na
stomach	normal	normal	soft	smooth	yellowish	normal	smooth	soft	na	na	na	na	na	na	na	na
intestine	greenish	enlarged	flabby	rough	normal	reduced	rough	hardened	reddish	enlarged	ni	hemorrhagic	na	na	na	na

na: samples not available; ni: aspect not identified.

Table S3: Results of yellow fever virus (RTqPCR) and Human Herpesviruses (qPCR) molecular investigation in *Callithrix penicilatta*

Marmosets/investigated viruses (and Cq values when samples were positive)

sample	**1342**		**1343**		**1344**		**1345**	
	HHV-1/2	HHV-1	HHV-1/2	HHV-1	HHV-1/2	HHV-1	HHV-1/2	HHV-1
Tongue	+ / 27.6	23	na	na	na	na	na	na
Kidney	+ / 31	nt	+ / 34.3	nt	+ / 30.5	nt	+ / 33.2	nt
Liver	+ / 34.4	36,5	+ / 37.8	35,7	+ / 31.9	29	+ / 34.3	34,6
Lung	+ / 35.2	33,9	+ / 37.5	36,8	+ / 33.8	34,3	+ / 29.9	29,4
Testicle	na	nt	+ / 35.2	nt	+ / 32	nt	+ / 29.3	nt
Heart	+ / 26.5	nt	+ / 34	nt	+ / 30	nt	+ / 26	nt
Intestine	+ / 32.5	nt	+ / 31.5	nt	+ / 29	nt	na	na
Bladder	na	nt	+ / 31	nt	na	na	na	na
Spleen	na	nt	na	na	+ / 34.7	nt	na	na

YFV: yellow fever virus; HHV-1/2: human alphaherpesvirus 1 and 2; HHV-3: Varicella zoster virus/human alphaherpesvirus 3; +: positive (for Cq values see Table S3); -: negative; na: sample not available. nt: sample was not tested. Cq:cycle threshold in real-time PCR

#PV358090
CGC GTC CAC CTC CTC GAC GAT GCA GTT TAC CGT CGT CCC GTA CCG GTG GAA CGC CTC CAC CCG CGA GGG GTT GTA CTT GAG GTC GGT GGT GTG CCA GCC
CCG GCT CGT GCG GGT CGC GGC GTT GGC CGG TTT CAG CTC CAT GTC GGT CTC GTG GTC GTC CCG GTG AAA CGC GGT GGT CTC CAG GTT GTT GCG CAC GTA
CTT GGC CGT GGA CCG ACA GAC CCC CTT GGC GTT GAT CTT GTC GAT CAC CTC CTC GAA GGG GAC GGG GGC GCG GTC CTC AAA GAT CCC CAT AAA CTG GGA
GTA GCG GTG GCC GAA CCA CAC CTG CGA AAC GGT GAC GTC TTT GTA GTA CAT GGT
#KU310663.1
... ... G.. ... ... G.. ... ... A.. ... ... C..
... ... C.. G.. .A. C.. C.. ... C.. G.. ... ...T ... ... C.. ... ...
... ...
... ...
#MF510364.1
... ... G.. ... ... G.. ... ... A.. ... ... C..
... ... C.. G.. .A. C.. C.. ... C.. G.. ... ...T ... ... C.. ... ...
... ... G.. G.. ... ... A.. ... ... A.. ... ... T.. ... ...
... ... C.. ... C.. ... ...
#MF510367.1
... ... G.. ... ... G.. ... ... A.. ... ... C..
... ... C.. G.. .A. C.. C.. ... C.. G.. ... ...T ... ... C.. ... ...
... ... G.. G.. ... ... A.. ... ... A.. ... ... T.. ... ...
... ... C.. ... C.. ... ...
#PP100088.1
... ... G.. ... ... G.. ... ... A.. ... ... C..
... ... C.. G.. .A. C.. C.. ... C.. G.. ... ...T ... ... C.. ... ...
... ... G.. G.. ... ... A.. ... ... A.. ... ... G.. T.. ... ...
... ... C.. ... C.. ... ...
#MH790555.1
... ... G.. ... ... G.. ... ... A.. ... ... C..
... ... C.. G.. .A. C.. C.. ... C.. G.. ... ...T ... ... C.. ... ...
... ... G.. G.. ... ... A.. ... ... A.. ... ... G.. T.. ... ...
... ... C.. ... C.. ... ...
#MH999842.1
... ...
... ...
... ...
... ...
#MN159381.1
... ...
... ...
... ...
... ...
#OR833069.1
... ...
... ...
... ...
... ...
#MH160368.1
... ...
... ...
... ...
... ...
#MG999850.1
... ...
... ...
... ...
... ...
#MH999849.1

```
... ... ... ... ... ... ... ... ... ... ... ... ... ... ... ... ... ... ... ... ... ... ... ... ... ... ... ... ... ... ... ... ...
... ... ... ... ... ... ... ... ... ... ... ... ... ... ... ... ... ... ... ... ... ... ... ... ... ... ... ... ... ... ... ... ...
... ... ... ... ... ... ... ... ... ... ... ... ... ... ... ... ... ... ... ... ... ... ... ... ... ... ... ... ... ... ... ... ...
... ... ... ... ... ... ... ... ... ... ... ... ... ... ... ... ... ...
#MG999847.1
... ... ... ... ... ... ... ... ... ... ... ... ... ... ... ... ... ... ... ... ... ... ... ... ... ... ... ... ... ... ... ... ...
... ... ... ... ... ... ... ... ... ... ... ... ... ... ... ... ... ... ... ... ... ... ... ... ... ... ... ... ... ... ... ... ...
... ... ... ... ... ... ... ... ... ... ... ... ... ... ... ... ... ... ... ... ... ... ... ... ... ... ... ... ... ... ... ... ...
... ... ... ... ... ... ... ... ... ... ... ... ... ... ... ... ... ...
#OP431901.1
... ... ... ... ... ... ... ... ... ... ... ... ... ... ... ... ... ... ... ... ... ... ... ... ... ... ... ... ... ... ... ... ...
... ... ... ... ... ... ... ... ... ... ... ... ... ... ... ... ... ... ... ... ... ... ... ... ... ... ... ... ... ... ... ... ...
... ... ... ... ... ... ... ... ... ... ... ... ... ... ... ... ... ... ... ... ... ... ... ... ... ... ... ... ... ... ... ... ...
... ... ... ... ... ... ... ... ... ... ... ... ... ... ... ... ... ...
#KX791867.1
... ... ... ... ... ... ... ... ... ... ... ... ... ... ... ... ... ... ... ... ... ... ... ... ... ... ... ... ... ... ... ... ...
... ... ... ... ... ... ... ... ... ... ... ... ... ... ... ... ... ... ... ... ... ... ... ... ... ... ... ... ... ... ... ... ...
... ... ... ... ... ... ... ... ... ... ... ... ... ... ... ... ... ... ... ... ... ... ... ... ... ... ... ... ... ... ... ... ...
... ... ... ... ... ... ... ... ... ... ... ... ... ... ... ... ... ...
```

Figure S1: Nucleotide alignment of the sequenced viral fragment along with HSV sequences from the database

References

Domingo C, Patel P, Yillah J, Weidmann M, Méndez JA, Nakouné ER, et al. Advanced yellow fever virus genome detection in point-of-care facilities and reference laboratories. J Clin Microbiol. 2012 Dec;50(12):4054–60.

Oliveira DB, Candiani TM, Franco-Luiz APM, Almeida GMF, Abrahão JS, Rios M, Coimbra RS, Kroon EG. Etiological agents of viral meningitis in children from a dengue-endemic area, Southeast region of Brazil. J Neurol Sci. 2017 Apr 15;375:390-394. doi: 10.1016/j.jns.2017.02.025. Epub 2017 Feb 16. PMID: 28320174.

Orle KA, Gates CA, Martin DH, Body BA, Weiss JB. Simultaneous PCR Detection of Haemophilus ducreyi, Treponema pallidum, and Herpes Simplex Virus Types 1 and 2 from Genital Ulcers. Vol. 34, JOURNAL OF CLINICAL MICROBIOLOGY. 1996.

Rezende IM, Alves PA, Arruda MS, Gonçalves AP, Oliveira GFG, Pereira LS, et al. Yellow fever virus genotyping tool and investigation of suspected adverse events following yellow fever vaccination. Vaccines (Basel). 2019 Dec 1;7(4).

Weidmann M, Meyer-König U, Hufert FT. Rapid detection of herpes simplex virus and varicella-zoster virus infections by real-time PCR. J Clin Microbiol. 2003 Apr 1;41(4):1565–8.
